# Buprenorphine Maintenance for Opioid Dependence in Public Sector Healthcare: Benefits and Barriers

**DOI:** 10.17352/2455-3484.000008

**Published:** 2015-08-03

**Authors:** Laura G. Duncan, Sonia Mendoza, Helena Hansen

**Affiliations:** 1 University of California, San Francisco; 2 New York University; 3 The Nathan S. Kline Institute for Psychiatric Research

**Keywords:** Buprenorphine, Addiction, Public healthcare, Low-income, Opioids

## Abstract

**Background:**

Since its U.S. FDA approval in 2002, buprenorphine has been available for maintenance treatment of opiate dependence in primary care physicians’ offices. Though buprenorphine was intended to facilitate access to treatment, disparities in utilization have emerged; while buprenorphine treatment is widely used in private care setting, public healthcare integration of buprenorphine lags behind.

**Results:**

Through a review of the literature, we found that U.S. disparities are partly due to a shortage of certified prescribers, concern of patient diversion, as well as economic and institutional barriers. Disparity of buprenorphine treatment dissemination is concerning since buprenorphine treatment has specific characteristics that are especially suited for low-income patient population in public sector healthcare such as flexible dosing schedules, ease of concurrently treating co-morbidities such as HIV and hepatitis C, positive patient attitudes towards treatment, and the potential of reducing addiction treatment stigma.

**Conclusion:**

As the gap between buprenorphine treatment in public sector settings and private sector settings persists in the U.S., current research suggests ways to facilitate its dissemination.

## Introduction

Upon FDA approval in 2002, buprenorphine became the first opioid medication in the U.S. since the 1914 Harrison Act that could be used for opiate dependence maintenance treatment in primary care physicians’ offices. This shift promoted integration of opiate dependence treatment into general medicine and some suggested that buprenorphine would attract new patients by providing an alternative to highly regulated methadone clinics [1]. Buprenorphine maintenance treatment implementation was intended for private practice treatment and current rates show that buprenorphine treatment does, in fact, primarily take place in private practices [2–5]. Buprenorphine is a partial opiate agonist with a limited ability to suppress breathing compared to methadone which is a complete agonist, and is primarily available in the U.S. under two different formulations known as Suboxone (buprenorphine/naloxone) and Subutex (buprenorphine) [6]. Buprenorphine offered a potential harm reduction tool for low-income patients with medical co-morbidities and for those at high risk for HIV, hepatitis C, and opiate overdose [6]. In this article, we argue that buprenorphine maintenance treatment is especially suited for implementation in U.S. public hospital and other government funded non-profit settings where vulnerable populations are primarily served.

Although there has not been a recent national representative demographic study in almost a decade, the latest, most complete U.S. based report from 2006 found that buprenorphine patients are Caucasian, are employed full time, and are seeking treatment for heroin or prescription opioid dependence [7]. Most buprenorphine patients were treated in private physician practices [7,8], and paid out-of-pocket [9] or were privately insured [10]. A study mapping buprenorphine prescriptions in New York City, the U.S. city with the largest opiate dependent population, confirmed higher prescription rates in high-income residential areas with low percentages of African American and Hispanic residents [11].

Treatment rate disparities have been fueled by the focus of buprenorphine marketing on the private sector [12] and by the perception that office-based buprenorphine treatment is most appropriate for employed, and therefore “stable,” patients [14,15]. Buprenorphine has been increasingly prescribed by primary care physicians; primary care physicians compose 63.5% of buprenorphine maintenance treatment providers in 2013 [5]. Despite an increase in buprenorphine maintenance providers, Stein et al found that 43% of U.S. counties have zero buprenorphine providers [15].

Buprenorphine’s comparable effectiveness to methadone in treating opioid addiction [16] and its tested suitability for varying therapeutic settings should be highlighted to promote implementation in public healthcare settings [17]. Buprenorphine maintenance treatment has additional characteristics that make it useful in the public sector, such as: 1) enhanced accessibility due to multiple venues for treatment, 2) flexible dosing that requires less institutional oversight than methadone, 3) demonstrated effectiveness among populations that heavily rely on public healthcare systems, such as the formerly incarcerated, and the homeless, 4) the potential to treat co-morbid chronic conditions prevalent among opiate dependent people such as HIV, and 5) the potential to lessen the stigma correlated with drug dependency among low income patients and ethnic minorities who already experience other forms of culturally defined social stigmatization [18,19]. This accumulated data can be used to improve the accessibility of buprenorphine as a first line treatment for heroin and opioid dependence for patients in public clinics.

## Causes of Treatment Disparities

The Drug Addiction Treatment Act of 2000 allowed for qualified physicians to seek certification to become waivered buprenorphine prescribers. However, only 3% of primary care physicians have buprenorphine waivers; [20] as of 2011, only 7% of U.S. counties had 20 or more buprenorphine providers [5]. Thomas et al. [21] found that while two-thirds of addictions specialists treated patients with buprenorphine, fewer than 10% of non-addiction specialist psychiatrists prescribed it. Many physicians feel they lack institutional support, experience, and training for themselves and clinic staff, and feel that the required 8-hour buprenorphine certification training is insufficient [22]. Physicians also reported inadequate institutional support as a major barrier to prescribing buprenorphine [23,24]. In the U.S., where addiction has historically been treated in specialty settings, many primary care providers perceive themselves as unprepared to discuss drug use with patients [25], even if they have already been treating known opioid users [22]. In a recent study regarding the barriers to buprenorphine maintenance treatment by family practitioners, physicians stated that their main barriers were inadequately trained staff and insufficient time as well as lack of knowledge, difficult patient population, and mistrust of opioid dependent patients [26]. Some physicians report concern about deception, suspicion of patient reported withdrawal symptoms [27], or worry that such patients would be disproportionately late to appointments [22]. Negative provider attitudes can also affect buprenorphine treatment rates; Krull et al. [28] found that directors of addiction treatment programs serving homeless patients generally had negative attitudes towards buprenorphine use, indicating a need for education of public service providers about the clinically efficacy of buprenorphine.

Correspondingly, physicians’ positive attitudes toward opioid maintained patients are associated with their willingness to treat them [29]. Cunningham et al. [30] found that physicians in primary care programs were more likely to express interest in prescribing buprenorphine than those in specialty care, suggesting opportunities for expansion of primary care based buprenorphine treatment. A recent study found that increasing the number of buprenorphine providers proportionally increased the number of buprenorphine treatment, suggesting that the current paucity of buprenorphine providers is limiting treatment opportunity [31].

Risk of buprenorphine diversion has also been cited as a major reason to not offer buprenorphine in public healthcare settings. A 2014 analysis of the factors associated with buprenorphine noncompliance found that use of benzodiazepines and psychiatric co-morbidities were associated with buprenorphine diversion [32]. The co-prescription and use of benzodiazepines with buprenorphine is harmful as both are respiratory suppressants and increase the risk of overdose [33,34]. As psychiatric co-morbidities and poly-substance dependence and abuse is common among opiate dependent patients [35], this could confound reasons for which providers without addiction medicine training or resources do not offer buprenorphine maintenance treatment.

The current profile of an illicit buprenorphine user is that of an experienced opioid user, having a history of snorting opioids, and identifying as Caucasian [36]. Patients in private practice are more likely to fit this profile than public sector patients who are more likely to be heroin users and to identify as African American or Hispanic. Physicians may not be likely sources for diverted buprenorphine since illicit buprenorphine users report obtaining the medication from a dealer, family member or friend, but not directly from a physician [37]. Primary reasons given for using diverted buprenorphine or injecting the medication were to suppress withdrawal symptoms and to modify a perceived inadequate dose [38,39]. While unemployment status was associated with increased risk of using diverted buprenorphine, an analysis found that receiving disability benefits decreased risk of using diverted buprenorphine [37], pointing to financial stability and social services as counteracting buprenorphine diversion. Illicit buprenorphine use is correlated with better treatment outcomes in primary care buprenorphine programs [40], and again suggests that a proportion of illicit buprenorphine users are attempting self-medication. Additionally, a social network analysis of buprenorphine diversion found that increasing access to providers reduced diversion rates [37].

Buprenorphine cost, reimbursement, and insurance coverage are also barriers to treatment in a public setting [7]. Ducharme and Abraham found in their 2008 analysis of the incorporation of buprenorphine maintenance treatment that government owned, non-profit programs were less likely to adopt buprenorphine than private, for profit programs [41]. Veteran Administration hospital dispensing data calculated that the cost of 6 months of buprenorphine care is comparable to that of methadone care over the same time span [42]. However, buprenorphine is excluded from most private health insurance plans or placed on the highest-tier formulary in order to control overall escalating prescription costs [43], while Medicaid coverage varies by state and is subject to restrictions. Office-based buprenorphine treatment has been shown to attract new patient populations [1,7], and Medicaid recipients are one of the fastest growing groups interested in the treatment [44]. Because substance abuse treatment without medication leads to greater relapse-related expenses and higher mortality, Medicaid coverage for buprenorphine treatment has been found to be cost effective in the long run [45] and changes in Medicaid reimbursement processes may lessen the gap between private and public sector treatment.

## Benefits of Integration into Public Sector

Multiple studies and reviews have established the effectiveness of buprenorphine for heroin [46] and prescription opioid dependence treatment [47], demonstrating a comparable effectiveness, safety, and treatment retention to methadone [16,48,49]. Buprenorphine has also demonstrated high patient satisfaction ratings [49] and a relatively low side effect profile [48]. Due to its federal approval for office-based use, buprenorphine can potentially offer flexible dosing and treatment location options. Varying buprenorphine dosing schedules (weekly vs. thrice-weekly) are similarly effective [50] and may offer better outcomes for some patients who do not show improvement on standard low doses [51]. Buprenorphine home induction demonstrated feasibility and safety with low withdrawal symptoms and similar retention rates to in-clinic induction [52]. Home-based inductions may also be a timesaving method for clinics that are not able to accommodate patients in active withdrawal [53]. Multiple counseling structures can offer benefits, including varying the timing and type of counseling^54–56^ to accommodate patient needs.

Buprenorphine treatment has been especially beneficial for opioid dependent populations with medical co-morbidities, the formerly incarcerated and homeless patients. A New York City based study tracked heroin users over a year and identified buprenorphine as a valuable harm reduction tool for socially marginalized users [2]. Past incarceration has no effect on primary care buprenorphine treatment outcomes [54,55], and previously incarcerated patients on buprenorphine treatment had better adherence and similar retention rates than those on methadone treatment [56]. The San Francisco Department of Public Health piloted a program offering office-based buprenorphine to patients who were homeless, unemployed, or living in poverty and found positive patient impressions, good retention rates, a positive shift in provider practices, and a decline in opioid use [57]. No significant differences in retention rates were found due to age, ethnicity, employment, or housing status. In a separate study of homeless patients, buprenorphine treatment was also associated with obtaining housing [28].

Primary care buprenorphine treatment has been associated with not only the treatment of chronic medical problems, but also the identification of previously unrecognized illness [56]. Bonhomme et al found that ethnic minorities with dual diagnosis of psychiatric illness and substance abuse tend to access healthcare in primary care settings [59]. HIV-positive patients suffering from opioid dependence may also benefit from combination buprenorphine treatment not only for reducing opioid use [60], but also increasing initiation or maintenance on antiretroviral treatment and improved CD4 counts [61]. Buprenorphine treatment has also been correlated with decreased injection drug use and lowering HIV risk behaviors [62]. Turner et al found that New York State physicians in clinics providing HIV care and physicians with experience treating intravenous drug users expressed more interest in providing buprenorphine than other physicians [29], making these settings a potential site for buprenorphine treatment integration.

An important facilitator to expansion of care in different settings is patients’ positive attitude toward buprenorphine treatment in primary care. Surveyed patients were satisfied with concurrent treatment for other health problems since it reduced their total number of medical appointments [22]. Patients also found that treatment locations were more convenient and were removed from illicit drug markets that often predominate around and near methadone clinics [22,63]. Patients were also satisfied with developing patient-provider relationships with primary care physicians and clinic staff [46] and favored “patient-focused” treatment where they felt they were offered autonomy, support, and trust from their provider [63]. A market survey at a South Bronx primary care clinic showed that there was high interest in buprenorphine as a mode of treatment for first time, low-income, substance abuse patients [64]. Additionally, patients who had previous experiences with both methadone and buprenorphine treatments preferred buprenorphine when readmitted to treatment, indicating that buprenorphine was a more attractive alternative to methadone [65].

In a review of the social stigma associated with substance dependence compared to mental illness, Schomerus et al found that drug users carried a greater burden of stigma and were seen as irrational, dangerous, and worthy of social rejection [66]. Methadone programs similarly are often marked by stigma due to punitive approaches to patients, staff characterization of patients as criminal or “dirty,” and barriers to social reintegration such as limited dispensing hours that coincide with work hours [57]. Providers holding negative views of methadone treatment for opiate dependence anticipated that office-based buprenorphine would reduce the stigma of drug dependence by removing addiction treatment from specialty clinics [22,47]. Stigma reduction is especially important for low income and ethnic minority patients who confront multiple sources of social marginalization in the U.S. Fareed et al. [51] report that patients felt more like a routine medical patient than an addiction patient when treated in a primary care buprenorphine clinic. Chiefly, positive patient-provider relationships associated with positive addiction treatment outcomes can be forged in primary care settings; such positive relationships are defined as consisting of organizational access, visit-based continuity, and knowledge of the patient as a “whole person” including an understanding of patient’s responsibilities, values, and beliefs outside of the clinic [67].

## Discussion

Although buprenorphine treatment rates in the public sector settings lag behind rates in private practice settings in the U.S. [41], there are considerable benefits of buprenorphine treatment in public settings. Concerns about diversion may have thwarted promotion of buprenorphine among low-income patients, but increased access to treatment may reduce the use and trade of non-prescribed buprenorphine. Although methadone treatment has long been a successful, cost-effective treatment for opioid dependence, buprenorphine is an attractive alternate and supplemental treatment option. While we described buprenorphine as adequate for public sector settings, negative prescriber attitudes, a shortage of certified buprenorphine prescribers, and economic barriers such as high cost of the medication and lack of insurance coverage must first be addressed to increase access.

Examples of local and regional initiatives that have addressed these concerns include the inclusion of buprenorphine coverage in state Medicaid formularies, state level media campaigns to increase physician and pharmacy awareness and adoption of buprenorphine [41,68], and prescriber support networks that partner with experienced prescribers for consultation. Such networks have been linked to the high rate of buprenorphine utilization in public clinics that have participated in federally funded clinical trials in which professional support was a feature of the study design [69–71].

The relationship and the association between diversion and adherence to buprenorphine treatment or positive treatment recoveries is a complicated one that does not always determine successful buprenorphine maintenance treatment and therefore should not be a barrier to implementation into public hospital settings.

As opioid use continues to be a public health concern in the U.S., buprenorphine treatment options need to be expanded in public sector settings. In particular, public sector physicians should be supported through professional buprenorphine mentoring networks, and encouraged to prescribe buprenorphine with higher insurance reimbursements as well as local institutional incentives for public clinic doctors to prescribe. Reimbursement incentives are justifiable on the grounds that buprenorphine allows for cost effective integrated care for a population with high levels of comorbidity and hospital readmission rates. Prescriber fears of diversion should be assuaged with data about the decrease of buprenorphine diversion when access to medically supervised buprenorphine is increased. Finally, potential buprenorphine patients should be provided with information about buprenorphine’s reduction of relapse rates, the advantages of office based buprenorphine for treatment of comorbidities, and the reports of prior buprenorphine patients that find buprenorphine treatment administratively flexible and less burdened with stigma.
